# Prospective evaluation of serum biomarker levels and cartilage repair by autologous chondrocyte transplantation and subchondral drilling in a canine model

**DOI:** 10.1186/ar2709

**Published:** 2009-05-26

**Authors:** Korakot Nganvongpanit, Peraphan Pothacharoen, Patama Chaochird, Kasisin Klunklin, Kanawee Warrit, Jongkolnee Settakorn, Nuttaya Pattamapaspong, Sirichai Luevitoonvechkij, Olarn Arpornchayanon, Prachya Kongtawelert, Dumnoensun Pruksakorn

**Affiliations:** 1 Bone and Joint Research Laboratory, Department of Veterinary Bioscience and Public Health, Faculty of Veterinary Medicine, Chiang Mai University, Kanklongchonpratan Road, Chiang Mai, 50100, Thailand; 2 Thailand Excellence Centre for Tissue Engineering, Department of Biochemistry, Faculty of Medicine, Chiang Mai University, Suthep Road, Chiang Mai, 50200, Thailand; 3 Musculoskeletal Research Laboratory, Department of Orthopedics, Faculty of Medicine, Chiang Mai University, Suthep Road, Chiang Mai, 50200, Thailand; 4 Department of Companion Animals and Wild Life, Faculty of Veterinary Medicine, Chiang Mai University, Kanklongchonpratan Road, Chiang Mai, 50100, Thailand; 5 Department of Pathology, Faculty of Medicine, Chiang Mai University, Suthep Road, Chiang Mai, 50200, Thailand; 6 Department of Radiology, Faculty of Medicine, Chiang Mai University, Suthep Road, Chiang Mai, 50200, Thailand

## Abstract

**Introduction:**

The purpose of this study was to evaluate serum chondroitin sulfate (CS) and hyaluronic acid (HA) levels and the capability of cartilage repair of full-thickness cartilage defects after treatment with two different fundamental surgical techniques: autologous chondrocyte transplantation (AC) and subchondral drilling (SD).

**Methods:**

A 4-mm-diameter full-thickness cartilage defect was created in each of 10 skeletally mature male outbred dogs. The dogs were randomly separated into two groups. Groups A and B were treated with AC and SD, respectively. An evaluation was made at the 24th week of the experiment. Serum was analyzed prospectively – preoperatively and at 6-week intervals – for CS and HA levels by enzyme-linked immunosorbent assay (ELISA) and ELISA-based assays, respectively.

**Results:**

The cartilage repair assessment score (median ± standard deviation) of group A (9.5 ± 2.5) was significantly higher than that of group B (2.5 ± 1.3) (*P *< 0.05). Group A also demonstrated a better quality of hyaline-like cartilage repair. Prospective analysis of serum WF6 and HA levels between the two groups did not show any significant difference. Serum WF6 levels at the 24th week of the experiment had a negative correlation (*r *= -0.69, *P *< 0.05) with the cartilage repair assessment score, whereas serum HA levels tended to correlate positively (*r *= 0.46, 0.1 <*P *< 0.05).

**Conclusions:**

AC treatment provides superior results to SD treatment, according to morphology, histology, and cartilage marker levels. AC treatment demonstrated a smoother surface, less fissure, better border integration, and a more reliable outcome of repairing cartilage. Moreover, a decreasing level of serum WF6, which correlated with good quality of the repairing tissue at the end of the follow-up period, was found predominantly in the AC group. Serum WF6 therefore should be further explored as a sensitive marker for the noninvasive therapeutic evaluation of cartilage repair procedures.

## Introduction

Due to a lack of effective monitoring methods after treatment, the optimal treatment for cartilage lesions has not been established. Although cartilage repair procedures – based on marrow-stimulating techniques such as subchondral drilling (SD), microfracture, and abrasion chondroplasty – provided initially favorable results, inferior fibrocartilage healing could lead to osteoarthritis in the long term [1]. Over the past decade, autologous chondrocyte transplantation (AC) has challenged previous techniques. Hyaline-like cartilage tissue was produced after treatment, as reported in several studies [2-6]. To determine the quality of cartilage repair and predict the long-term results, a noninvasive method that can be objectively interpreted and that is available for long-term application would be of great assistance. Serum biomarkers, which have been widely studied in the treatment of many musculoskeletal diseases, might be one of the most promising tools that could be used for such purposes. Herein, we prospectively analyzed serum chondroitin sulfate (CS) and hyaluronic acid (HA) levels using monoclonal antibody WF6 and enzyme-linked immunosorbent assay (ELISA), respectively, accompanying the cartilage repair assessment of two distinct fundamental surgical techniques: cell-based therapy (AC) and marrow-stimulating technique (SD).

## Materials and methods

### Animal model

Ten skeletally mature adult male outbred dogs, each weighing approximately 11 to 15 kg, were used in the study. Prior to recruitment, their knee joints were investigated using magnetic resonance imaging to exclude animals with degenerative joint disease or other orthopedic problems. The experimental protocol was approved by the Faculty of Veterinary Medicine and the ethics committee of Chiang Mai University, Thailand.

Animals were randomly separated into two groups. Group A was treated with AC, and group B was treated with SD. All operations were performed on the knee joint, with the animal under anesthesia and in sterile conditions. A prophylactic antibiotic (cephalexin) and an anti-inflammatory drug (vedaprofen; Intervet, Bangkok, Thailand) were given intravenously in three doses over the course of a 24-hour period during and after surgery. Both groups of animals were operated on three times. Full-thickness cartilage defect creation and cartilage harvesting were performed simultaneously. AC or SD was carried out over the following 4 weeks. All animals underwent arthrotomy and core biopsy at the 24th week of the experiment (the 20th week after treatment) for final evaluation. All animals were kept for phase II experiments.

For the cartilage harvesting procedure, the right knee joint was opened by an anteromedial approach. A 4-mm-diameter articular cartilage defect was created at the medial side of the trochlea of the femur by means of a 4-mm-diameter dermal punch to outline the defect. A customized curette was used to scrape all cartilage to the zone of calcified cartilage in both groups. Chondrocytes were isolated from the shavings and cultured as described below. Animals were allowed unrestricted cage activity after surgery.

At the fourth week, group A (n = 5) was treated with AC. The cartilage defect was covered with a periosteal flap (with the cambium layer facing toward the bone), which had been harvested from the proximal tibia. The flap was sutured to the surrounding rim of the normal cartilage with interrupted 8-0 sutures, and the surrounding edge was sealed with fibrin glue (FibrinGluRAAS, Shanghai RAAS Blood Products Co. Ltd., Shanghai, China). Cultured chondrocytes in culture medium (2 × 10^6 ^cells per defect) were injected beneath the periosteal flap. Group B (n = 5) was treated with SD. The cartilage defect was treated by using a 1.2-mm drill to create three drill holes. Before the joint was closed, bleeding vessels were cauterized and the patella was relocated. The joint was closed and the knee immobilized by a bulky soft splint for 1 week and a partial splint for 1 additional week. The splints and stitches were removed at the end of the second week, after which all animals were allowed to ambulate normally until the end of the experiment [4-6]. Cartilage removal in both groups and the covering of the defect with periosteum in group A were performed under loupe visualization.

### Isolation and culture of chondrocyte

Articular cartilage chondrocytes were isolated from the femoral trochlea as previously described [7]. Briefly, chondrocytes were isolated by digestion with 0.2% type II collagenase for 16 hours and resuspended in Dulbecco's modified Eagle's medium (DMEM) (Gibco, now part of Invitrogen Corporation, Carlsbad, CA, USA) containing 100 units/mL penicillin and 100 units/mL streptomycin. Chondrocytes were plated in tissue culture flasks at 10^4 ^cells per square centimeter and cultured in DMEM/F-12 supplemented with 10% heat-inactivated fetal bovine serum (Invitrogen Corporation) at 37°C in a humidified atmosphere of 5% carbon dioxide and 95% air. After 10 days, when cells had reached subconfluence, the passage-1 cells were detached by treatment with 0.25% trypsin/1 mM ethylenediaminetetraacetic acid (EDTA) and plated at 5 × 10^3 ^cells per square centimeter. Chondrocytes from second passages were used in the following experiments.

### Biomarker assay

Serum was obtained from both groups. The first sample was started at the first date of the first week of the experiment before the first operation and the subsequent samples were taken every 6 weeks until the end of the experiment (a total of 24 weeks of experiment or 20 weeks of postoperative follow-up). Serum was centrifuged at 6,500 *g *for 10 minutes. Supernatants were stored at -80°C until assay.

### Competitive immunoassay using monoclonal antibody WF6

A mouse monoclonal antibody WF6 was raised against a shark cartilage aggrecan preparation [8], and a quantitative ELISA for the epitope recognized by monoclonal antibody WF6 was modified from a previous study [9]. The antibody was specific for intact CS chains and showed no interaction with other sulfated glycosaminoglycans, hyaluronan, or other polyanions, such as DNA, RNA, or dextran sulfate [9]. The standard used in the assay was shark cartilage aggrecan (A1 fraction) at concentrations of 19 to 10,000 ng/mL in 6% bovine serum albumin (BSA) in Tris-incubating buffer (0.1 M Tris HCl, pH 7.4, containing 0.15 M sodium chloride, 0.1% Tween 20 and 0.1% BSA). Diluted human serum samples (1:5 in 6% BSA-Tris-incubating buffer) were added to 1.5-mL plastic tubes containing an equal volume of WF6 (cell culture supernatant, 1:200 dilution in Tris-incubatingbuffer). They were incubated at 37°C for 1 hour and added to the microtiter plate, which was precoated with shark aggrecan (A1 fraction). Nonspecific protein binding was blocked with BSA. The plates were incubated at 37°C for 1 hour, the wells were washed, and peroxidase-conjugated anti-mouse IgM antibody (1:2,000) was added (100 mL/well in Tris-incubating buffer). The bound conjugate was detected by adding *ortho*-phenylenediamine (*o*-PD) substrate (100 mL/well in 0.05 M citrate buffer, pH 5.0). The reaction was stopped after 10 minutes with 50 mL/well of 4 M sulfuric acid, and absorbance was determined using a microplate reader at 492/690 nm. The concentration of WF6 epitope in supernatant samples was calculated by reference to a standard curve.

### Enzyme-linked immunosorbent assay-based assay for hyaluronic acid (HA) using biotinylated HA-binding proteins

Human serum samples or standard HA (HealonR) at various concentrations (19 to 10,000 ng/mL in 6% BSA-phosphate-buffered saline [BSA-PBS], pH 7.4) was added to 1.5-mL plastic tubes containing biotinylated HA-binding proteins (HABPs) prepared as described above (1:200 in 0.05 M Tris-HCl buffer, pH 8.6). The tubes were incubated at room temperature for 1 hour, and samples were added to the microplate, which was precoated with umbilical cord HA (100 mL/well of 10 mg/mL) and blocked with 1% BSA (150 mL/well). The plate was incubated at room temperature for 1 hour. The wells were washed, and peroxidase-conjugated antibiotin antibody (1:2,000 dilution), 100 mL/well in PBS, was added. The plate was incubated at room temperature for another hour. The detection of conjugated antibody was with *o*-PD substrate, and plate reading was carried out as described above. The concentration of HA in samples was calculated from the standard curve [8,10].

### Tissue preparation

At the 24th week of the experiment, repaired tissues were examined grossly and photographically. Normal cartilage and repaired cartilage were harvested by using 5-mm-diameter custom-made core biopsies. Tissue was placed in 10% neutral buffered formalin. Each sample was placed into 15% disodium EDTA decalcifying solution (pH 7.4) and shaken at 4°C. Decalcifying solution was changed three times each week for 4 weeks. The specimens were rinsed thoroughly, dehydrated, and embedded in paraffin at 60°C. Seven-micrometer-thick sections were prepared and were stained with hematoxylin and eosin and with Safranin O for microscopic examination of the repaired tissue [10].

### Histology evaluation

At the 24th week of the experiment, the quality of the repaired tissue was evaluated by two methods. Repaired cartilage was blindly evaluated by orthopedic surgeons intraoperatively. Lesions were graded for the extent of cartilage repair according to the cartilage repair assessment from the International Cartilage Repair Society (ICRS) Cartilage Injury Evaluation Package [11]. Histological sections from each animal were scored by a pathologist according to (a) surface, (b) matrix, (c) cell distribution, (d) cell population viability, (e) subchondral bone, and (f) cartilage mineralization, using the ICRS Visual Histological Assessment Scale [12].

### Statistical analysis

The general identification information and cartilage score assessment (ICRS Cartilage Injury Evaluation Package) of the two groups were analyzed by means of the Student *t *test. Quantitative data of serum WF6 and HA were analyzed by a nonparametric Wilcoxon rank sum test. All data were considered significant when *P *values were less than 0.05. The correlation between serum WF6 and HA levels and the cartilage score assessment was analyzed by means of the Kendall rank correlation coefficient test. All statistical analysis was performed with STATA software version 10.0 (StataCorp LP, College Station, TX, USA).

## Results

General information about all animals, including age, gender, weight, and basic biochemistry laboratory findings, is shown in Table 1. All 10 canines ambulated normally after splint removal, and there were no infections or complications during postoperative monitoring through the 24th week of the experiment. At the time of the final operation, all synovial tissue appeared normal. Joint fluid was within a normal range, with some slightly wetter or drier.

**Table 1:** Identification data of animals

Parameter	AC (n = 5)^a^	95% CI	SD (n = 5)^a^	95% CI	*P *value
Gender	Male	-	Male	-	-
Age (month)	40 ± 15	21.4–58.6	38 ± 16	18.1–57.9	0.844
Weight (kg)	14.1 ± 0.8	13.1–15.1	13.1 ± 2.1	10.5–15.7	0.356
BUN (mg/dL)	13.7 ± 3.1	9.9–17.5	14.3 ± 3.9	9.5–19.1	0.793
Cr (mg/dL)	1.1 ± 0.11	0.9–1.2	1.0 ± 0.1	0.8–1.2	0.581
AST (IU/L)	31.0 ± 13.5	14.2–47.8	31.0 ± 9.3	19.5–42.5	1.000
ALT (U/L)	20.0 ± 4.5	14.4–25.6	25.4 ± 5.9	18.1–32.7	0.142
Hemoglobin (g/dL)	13.8 ± 1.8	11.6–16.1	13.6 ± 1.9	11.2–15.9	0.817
White blood cells (/μL)	10,560.0 ± 1,705.3	8,442.6–12,677.4	9,870.0 ± 1,284.3	8,275.3–11,464.7	0.490

The two groups show similar baseline characteristics. ^a^Values are the mean ± standard deviation. AC, autologous chondrocyte transplantation; ALT, alanine aminotransferase; AST, aspartate aminotransferase; BUN, blood urea nitrogen; CI, confidence interval; Cr, creatinine; SD, subchondral drilling.

### Cartilage repair evaluation

**Figure 1:**
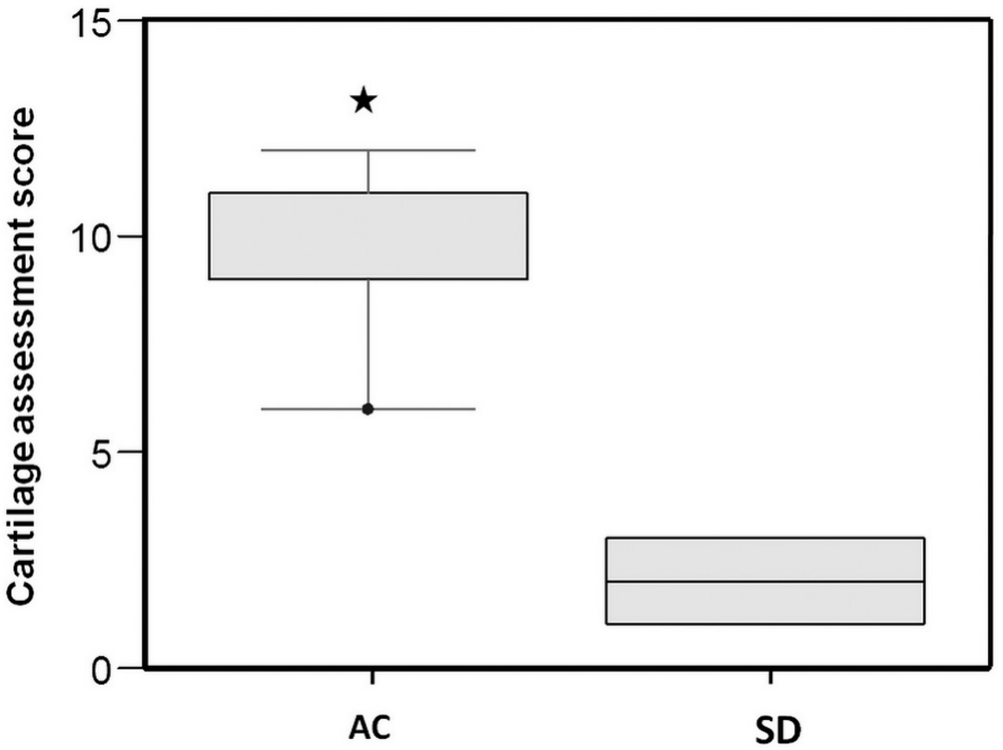
A comparison of Cartilage Repair Assessment Score (International Cartilage Repair Society) between autologous chondrocyte transplantation (AC) and subchondral drilling (SD). Boxes represent medians and interquartile ranges, between the 5th and 95th quartiles, with error bars. Cartilage repair assessment score is significantly higher for the AC group than the SD group (*P <0.05).

As shown in Table 2, for the AC group, dog number 1 (D1) shows normal repaired cartilage tissue; D2, D4, and D5 show nearly normal results; and D3 showed abnormal repair, with 50% filling of repaired cartilage and approximately half of the repaired cartilage integrated with the normal cartilage border. On the other hand, most of the SD group showed poorer results. D6, D8, and D9 presented total degeneration of the repair site, with further degeneration of the surrounding normal hyaline cartilage. D7 and D10 showed repairing tissue around the drilling holes, with velvet surface appearance and significant separation of the repair site (depending on the drilling hole) without continuity of the normal cartilage border. The scores of cartilage repair (mean ± standard deviation) were significantly higher in the AC group: 9.4 ± 2.3 for AC versus 2.0 ± 1.0 for SD (*P *< 0.05) (Figure 1).

**Table 2:** Morphological evaluation of the treatment using Cartilage Repair Assessment (ICRS Cartilage Injury Evaluation Package) [11]

Parameters	Autologous chondrocyte transplantation					Subchondral drilling				
		
	D1	D2	D3	D4	D5	D6	D7	D8	D9	D10
Degree of defect repair	4	2	2	4	3	0	3	2	1	3
Integration of border zone	4	3	1	4	3	0	0	0	0	0
Macroscopic appearance	4	4	3	3	3	1	0	0	0	0
Overall assessment	12	9	6	11	9	1	3	2	1	3
Grade	I	II	III	II	II	IV	III	III	IV	III

D1, D2, and so on refer to dog number 1, dog number 2, and so on. ICRS, International Cartilage Repair Society.

### Histology evaluation

**Figure 2:**
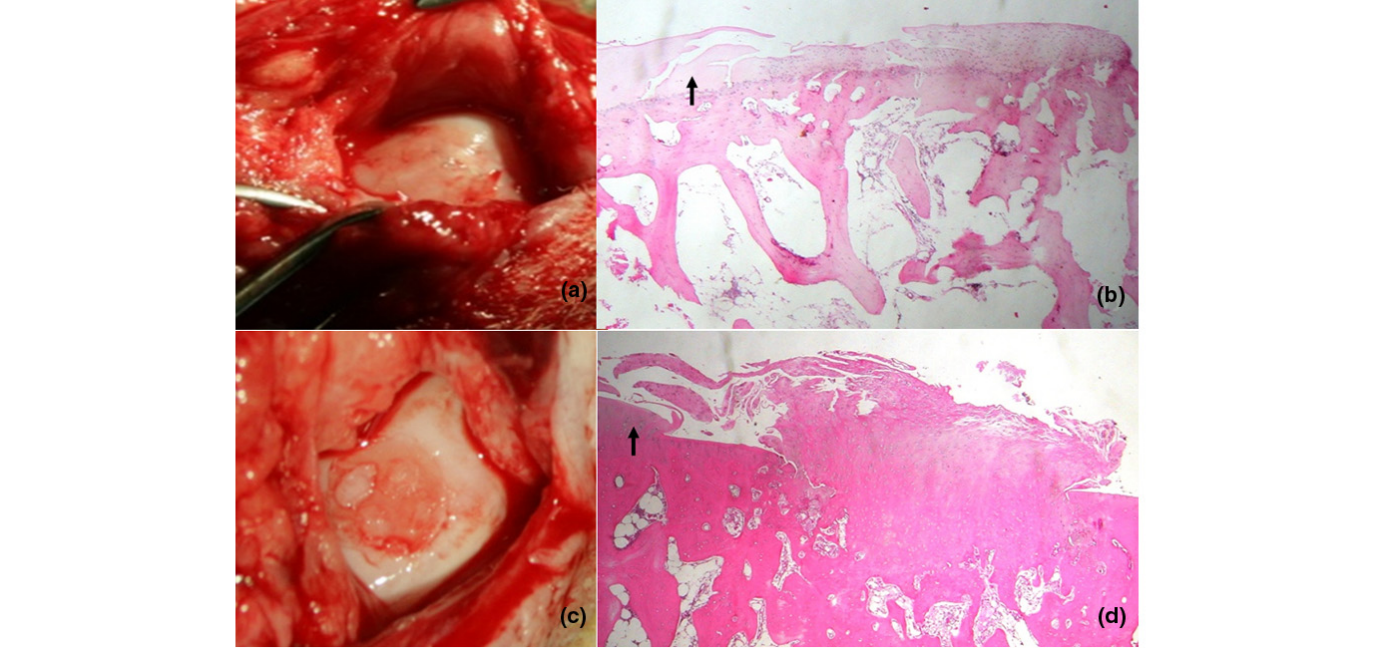
Intraoperation of repaired articular cartilage and low-power-field microscopic evaluation. **(a) **Articular defect fully filled with new cartilage tissue. **(b) **Flat cartilaginous proliferation and smooth surface compared with normal articular cartilage on the left-hand side (arrow) in the autologous chondrocyte transplantation treatment group. **(c) **Subchondral bone exposed with small intralesional cartilage island showing a velvet surface. **(d) **Exuberant fibrocartilaginous proliferation with irregular surface compared with normal articular cartilage on the left-hand side (arrow). (b, d) Stain: hematoxylin and eosin; original magnification: × 40.

**Figure 3:**
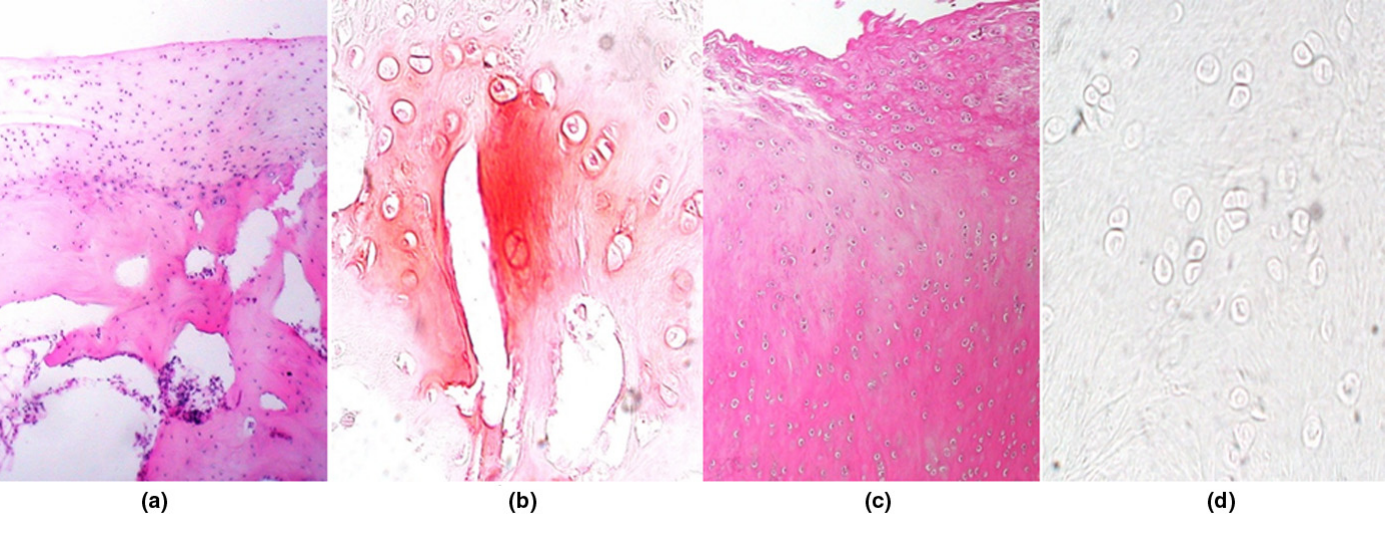
A high-power-field microscopic evaluation of cartilage histology with special staining. **(a) **Repaired cartilage from the autologous chondrocyte transplantation group (hematoxylin and eosin) demonstrates a cellular arrangement, **(b) **and glycosaminoglycan-containing extracellular matrix, which has been shown by positive staining of Safranin O. **(c) **Repaired fibrocartilage from the subchondral drilling group with fibroblast-like cells, **(d) **and without glycosaminoglycan matrix was found using Safranin O stain.

Cartilage tissue of the AC group demonstrated a more columnar arrangement of cell distribution and was mostly a mix of hyaline/fibrocartilage-like matrix. Despite an increasing cell population viability in the SD group, the matrix appeared predominantly in the fibrous tissue or fibrocartilage-like matrix, corresponding to the velvet surface repair in macroscopic appearance. Subchondral bone characterization and cartilage mineralization in AC showed a better remodeling result than that of SD (Figures 2 and 3; Table 3)

**Table 3:** Histological evaluation of cartilage treatment using ICRS histological assessment at the 20th week of follow-up after AC and SD treatments

Parameters	Autologous chondrocyte transplantation					Subchondral drilling				
		
	D1	D2	D3	D4	D5	D6	D7	D8	D9	D10
Surface	3	3	0	3	3	0	0	0	0	0
Matrix	2	3	2	2	2	1	1	2	2	1
Cell distribution	2	3	2	2	2	0	0	2	1	1
Cell population viability	3	2	2	3	2	3	3	3	3	3
Subchondral bone	2	3	2	3	2	2	2	2	2	2
Cartilage mineralization	0	3	0	3	0	0	0	0	0	0

D1, D2, and so on refer to dog number 1, dog number 2, and so on. AC, autologous chondrocyte transplantation; ICRS, International Cartilage Repair Society; SD, subchondral drilling.

### Serum cartilage marker evaluation

**Figure 4:**
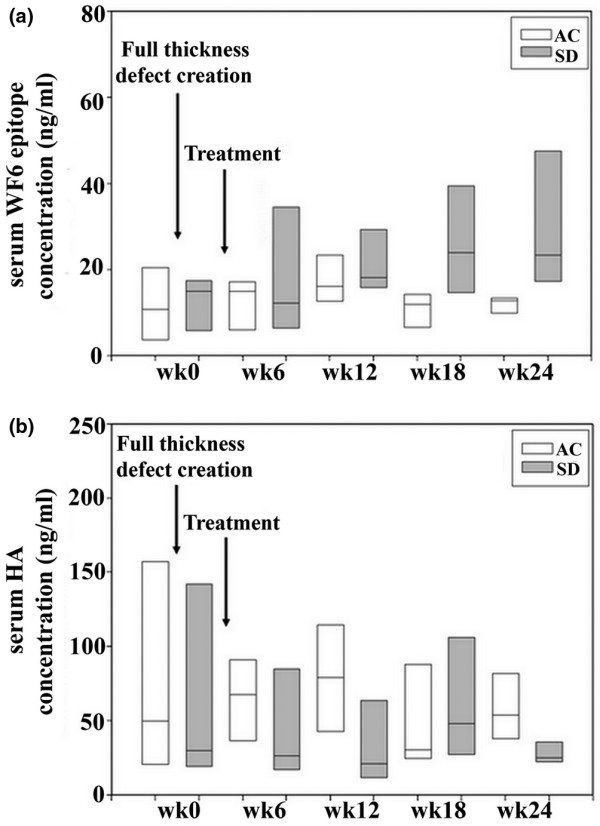
The prospective values of serum WF6 **(a)** and hyaluronic acid (HA) **(b)** concentration (n = 10). Boxes represent medians and data distribution. The results show no statistically significant difference of serum WF6 or serum HA level at any time point. AC, autologous chondrocyte transplantation; SD, subchondral drilling.

**Figure 5:**
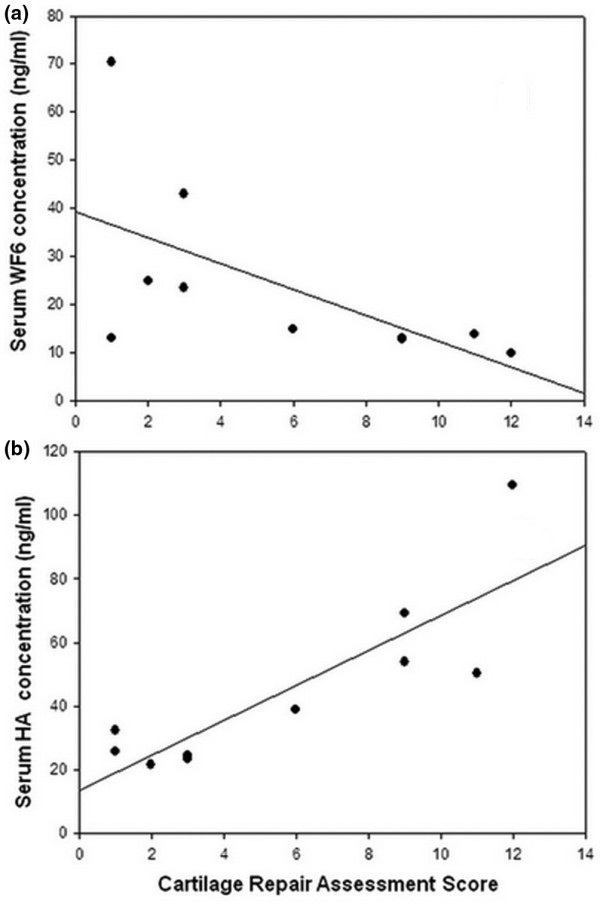
Analysis of the correlation between cartilage assessment scores at 20 weeks after treatment and levels of serum WF6 **(a)** and hyaluronic acid (HA) **(b)**. Correlation analysis was conducted using the Kendall rank correlation coefficient test. Serum WF6 level negatively correlates with the final assessment score (*r *= -0.69, *P *< 0.05), and serum HA level tends to positively correlate (*r *= 0.46, 0.1 <*P *< 0.05) with the cartilage repair assessment score.

Serum WF6 levels of all animals progressed to higher levels during the 6th and 12th weeks in comparison with the preoperative level and slightly decreased during the 18th and 24th weeks in the AC group; however, those changes did not demonstrate a statistically significant difference between the two treatment groups (Figure 4a). On the other hand, serum HA level presented more variability than serum WF6 level, and the result progressively decreased from preoperative levels for both treatment groups. Serum HA level also did not show a significant difference in prospective follow-up between the two groups (Figure 4b). By cross-sectional analysis of markers, serum WF6 level at the 24th week of the experiment showed a negative correlation with the cartilage repair assessment score (*r *= -0.69, *P *< 0.05), whereas serum HA level tended to present a positive correlation (*r *= 0.46, 0.1 <*P *< 0.05) (Figure 5).

## Discussion

We prospectively studied two distinct fundamental surgical techniques for cartilage treatment. For the AC group, cultured chondrocytes were injected under the periosteal flap sutured over the defect; this retains the injected cells, and the cambium layer participates in the process of chondrogenesis [6,13]. SD, a traditional cartilage defect treatment, was performed by penetrating the subchondral bone; this facilitates the migration of the injected cells, as first reported by Pridie in 1959. This method has been widely used for osteoarthritis and cartilage defect treatment since it is an easy surgical technique and improves the initial clinical outcome [14].

In this study, the AC technique demonstrated obviously better results than the SD technique, including smooth surface, border integration, and reliable tissue repair. The periosteum covering technique not only provides an initially smooth texture outline for the chondrocytes but also enhances differentiation and proliferative potential for the new repairing tissue, as has been reported previously [6,15]. The liquidity of the autologous chondrocyte solution can fill in any shape of defect, regardless of size and depth; therefore, the repairing tissue demonstrates good integration of normal cartilage border and fewer problems of cartilage fissure as opposed to SD techniques. The repairing process in the AC group doubly benefited from two factors – that is, chondrocytes and periosteum – so that in all cases the AC group demonstrated more reliable repair than the SD group did. AC techniques are more complicated and create longer surgical scars for surgical field exposure and periosteal harvesting, whereas SD can be finished in less time and with a smaller surgical incision. However, SD provided unreliable results in this study: two cases did not repair, and another showed a small colony of new cartilage repair in a large defect. In the other two cases, despite the repairing tissue filling the defect, poor-quality tissue was present around the drilling hole and was unable to integrate with the surrounding normal cartilage tissue. The SD technique could not control the number of cells, mesenchymal cell type, or flow direction of marrow on the repair site, leading to the poorer results in this experiment.

In current practice, because of limited sensitivity and ineffective methods of postoperative evaluation, the optimal treatment for cartilage lesions has not been established. Although favorable clinical and short-term histological outcomes have been reported from AC treatment since 1994 [3-6,16], some clinical studies did not show significant advantages of AC over other operations [16,17]. Mechanisms of the repairing process should be studied not only at the molecular level but also in terms of the time-dependent changes in cartilage repair in order to predict the long-term outcome of a particular surgical treatment. Cartilage markers are among the promising tools that are able to overcome such problems. Therefore, CS and HA levels in circulation after cartilage defect creation and surgical treatment were prospectively analyzed in this study.

CS is the major component of proteoglycan embedded within the fibrillar collagen network in articular cartilage. The highly sulfated structure leads to a large amount of hydration, which provides the compressive stiffness of articular cartilage [18]. For patients with anterior cruciate ligament injury, instability results in an initial increase of proteoglycan content and collagen breakdown of articular cartilage within 1 year of injury [19,20]. This finding corresponds with the increasing proteoglycan level in synovial fluid and serum detected by cartilage markers [21-23]. In this study, the monoclonal antibody WF6 was used to detect changes in serum CS levels. WF6 epitope level was variably expressed in the extracellular matrix of hyaline cartilage, which could characterize the releasing pattern of proteoglycans [8-10]. After cartilage defect creation and treatment, serum WF6 level tended to increase for the first 12 weeks of the experiment. This increased level agreed with the repairing period (within 3 months) of cartilage in previous reports of the canine model [4]. The level decreased to nearly baseline level after 12 weeks. In subgroup observation, from the 12th week, serum WF6 level in the SD group tended to increase (Figure 4a). Therefore, the poorer cartilage repair in the SD group should be an important contributing factor for proteoglycan turnover and could lead to secondary osteoarthritis. Moreover, the better cartilage repair assessment score was related to the lower level of serum WF6 (*r *= -0.69, *P *< 0.05) at the 24th week of the experiment, according to the correlation analysis. The slow rate of cartilage turnover reflects the balancing homeostasis of articular cartilage in good repair tissue. Thus, serum WF6 level could be a sensitive marker for determining the quality of repair tissue in this model.

HA plays the key role in immobilizing aggrecans in articular cartilage; this balances the tension and compressive resilience in the collagen network by its osmotic properties [24]. HA decreases the molecular size of the cartilage matrix and increases its proportion to the aggrecans by age-related change [25]. Serum HA has been studied as a biomarker of disease progression from the time that significantly increased levels were reported in cases of rheumatoid arthritis and progressive osteoarthritis compared with the normal population [8,26-28]. For acute injury models, several studies have demonstrated decreasing HA concentration in synovial fluid [29-32], but some reports did not show any relation to serum level [22,30]. The serum HA concentration of this prospective follow-up of full-thickness cartilage creation and treatment agrees with previous reports, in which it was not significantly different from baseline levels. Nevertheless, the release pattern of HA into circulation tends to decrease from baseline over time and never reaches normal levels. This should correlate with the reduction of synovial HA levels of previous acute injury models. Another interesting observation was the trend of positive correlation between cartilage repair assessment score and serum HA level (0.1 <*P *< 0.05). Here, the higher serum HA level might determine whether the joint returns to homeostasis of the intra-articular condition.

Sample size is an important factor that limits the power of interpretation. The current study model is different from the usual acute traumatic model, one that is anterior cruciate ligament-sacrificed and that has been used and presented as an obvious serum marker change in several studies. Full-thickness cartilage creation without instability could not create the generalized cartilage change similar to that of the traumatic model. That is probably an important effect, making any significant differences undetectable by serum biological markers. This study presents an important use of biomarkers as a tool for studying the postoperative release patterns of CS and HA. This result will provide fertile ground for further exploration of sensitive markers in such cartilage treatment procedures.

## Conclusions

The results of AC treatment are superior to those of SD treatment, according to morphology, histology, and cartilage marker level. AC treatment demonstrated a smooth surface, less fissure, good border integration, and a reliable outcome of repairing cartilage. Serum WF6 level could represent the quality of cartilage repair postoperatively, whereas serum HA level should be further studied for its circulating-kinetic pattern.

## Abbreviations

AC: autologous chondrocyte transplantation; BSA: bovine serum albumin; CS: chondroitin sulfate; D1 to 10: dog number 1 to 10; DMEM: Dulbecco's modified Eagle's medium; EDTA: ethylenediaminetetraacetic acid; ELISA: enzyme-linked immunosorbent assay; HA: hyaluronic acid; ICRS: International Cartilage Repair Society; o-PD: ortho-phenylenediamine; PBS: phosphate-buffered saline; SD: subchondral drilling.

## Competing interests

The authors declare that they have no competing interests.

## Authors' contributions

DP, OA, and KN carried out the design, operation, and coordination of the study and helped to draft the manuscript. PK and PP carried out the biochemistry assay and chondrocyte culture. KK, PC, and KW participated in operative anesthesia and preoperative and postoperative animal care. JS and NP participated in pathological and radiological interpretation. SL participated in statistical analysis. All authors read and approved the final manuscript.
